# Supplementary material for: PepMCP: a graph-based membrane contact probability predictor for membrane-lytic antimicrobial peptides
Source: Bioinformatics. 2026 Jun 26;42(7):btag453. doi: 10.1093/bioinformatics/btag453 (PMC13371751; doi:10.1093/bioinformatics/btag453)
Supplement: btag453_Supplementary_Data [file btag453_supplementary_data.pdf]

Supplementary Information for:

PepMCP: A Graph-Based Membrane Contact Probability Predictor for Membrane-Lytic Antimicrobial Peptides

Ruihan Dong[1,2], Tadsanee Awang[1], Qiushi Cao[1], Kai Kang[1,2], Lei Wang[1], Zefeng Zhu[1,2], and Chen Song[1,*]

[1]*Center for Quantitative Biology, Peking-Tsinghua Center for Life Sciences, Academy for Advanced Interdisciplinary Studies, Peking University, Beijing 100871, China*

[2]*Peking University–Tsinghua University–National Institute of Biological Sciences Joint Graduate Program, Academy for Advanced Interdisciplinary Studies, Peking University, Beijing 100871, China*

*E-mail: c.song@pku.edu.cn

Supplementary Figures

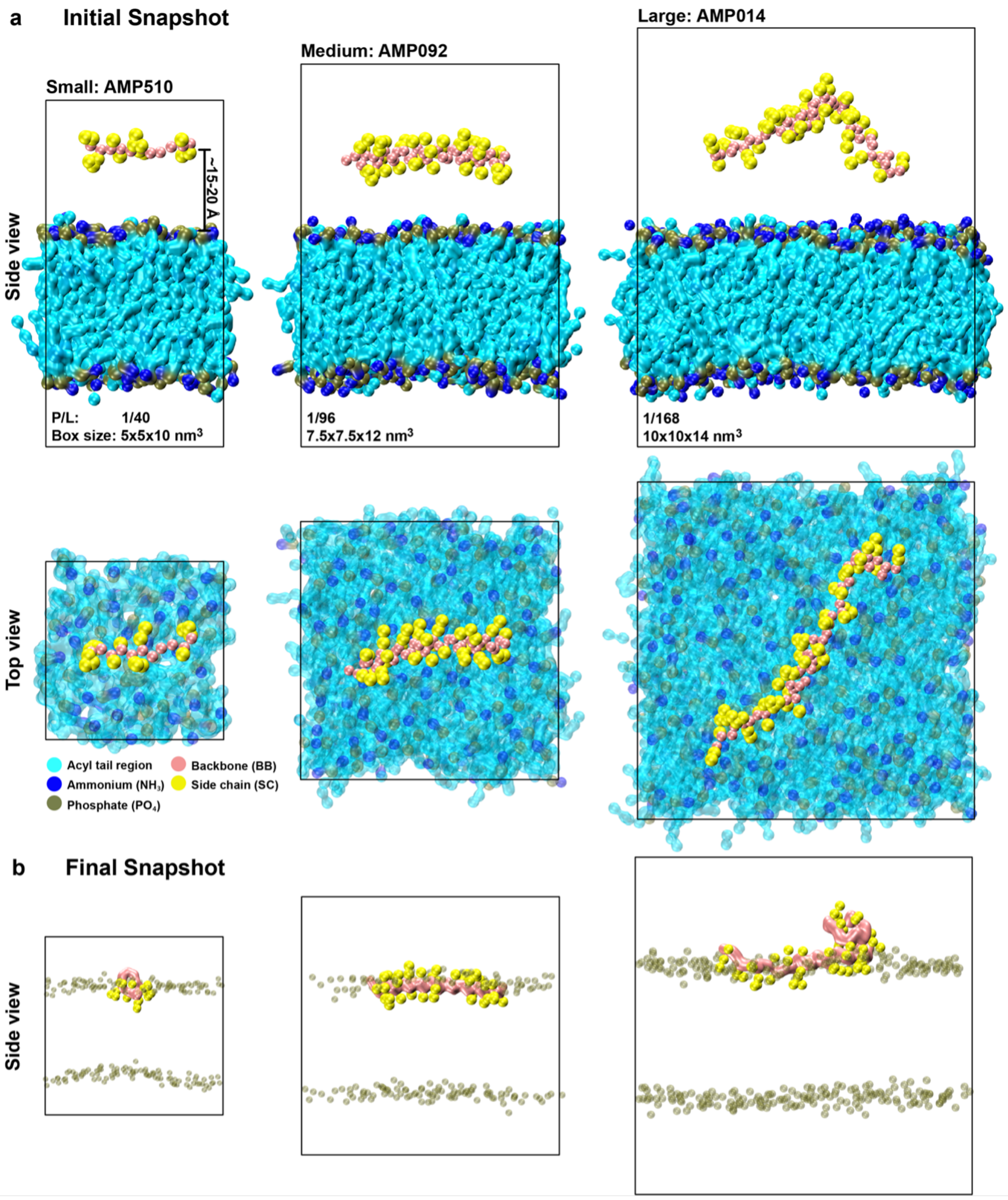

Figure S1: Coarse-grained (CG) simulation systems for peptide–membrane interactions. **a**. Initial side and top views of small, medium, and large simulation boxes showing the placement of peptides above the membrane, with the peptide center of mass positioned $\sim$15–20 Å from the membrane headgroup region. Corresponding peptide-to-lipid (P/L) ratios and box dimensions are indicated, along with representative peptide conformations within each simulation box. **b**. Final side view snapshots of each system. The cases for each system size were: small-AMP510 (synthetic peptide #14d), medium—AMP092 (MAP34-B), and large—AMP014 (prosthecin-1), respectively. The small and medium systems were at 2 μs, while the large was at 3 μs. Color scheme: lipid acyl tail region (cyan), ammonium headgroups (blue), phosphate headgroups (olive), peptide backbone (salmon), and side chains (yellow). Molecular structures were visualized using VMD version 1.9.4.

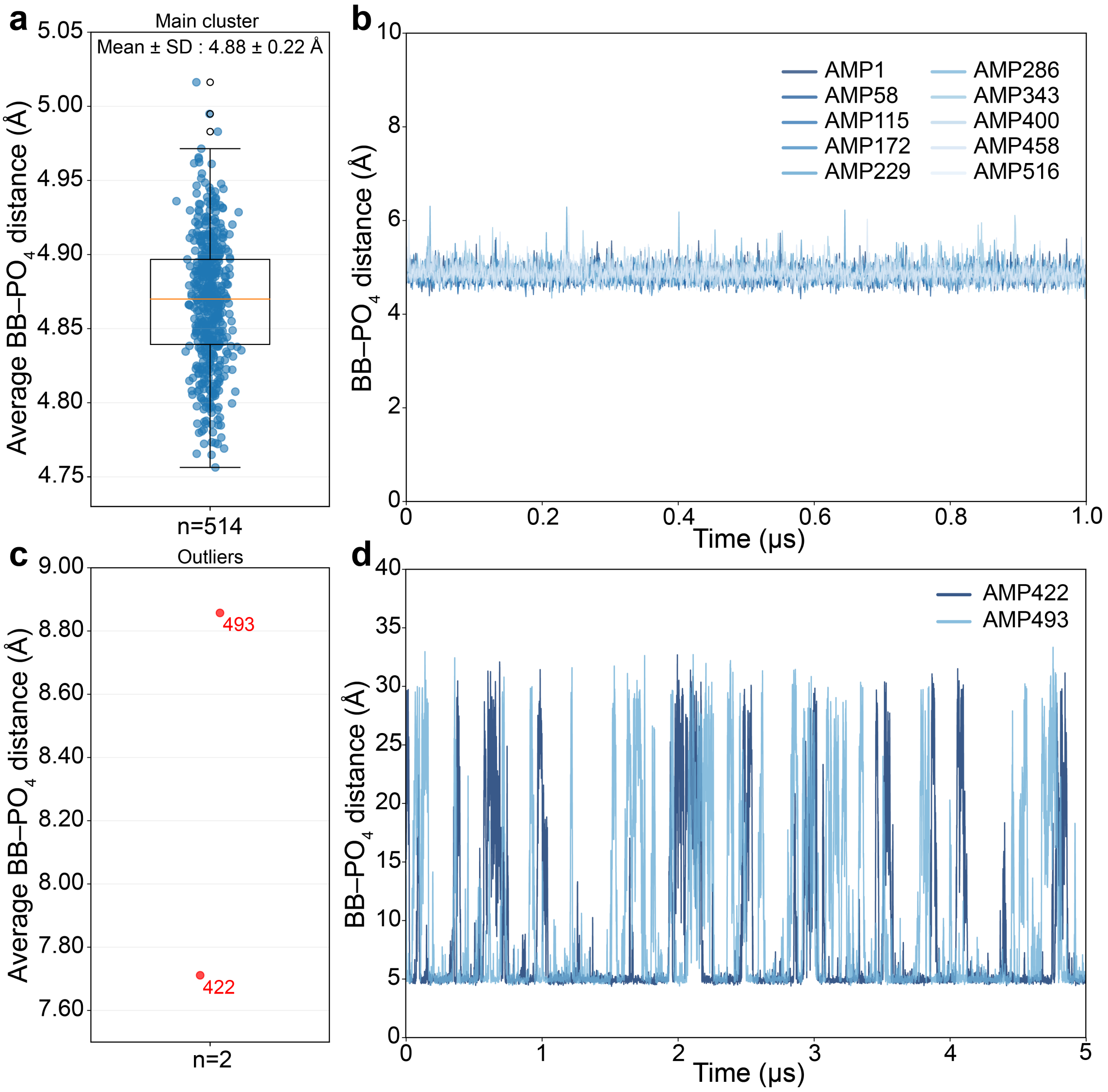

Figure S2: Binding of membrane-lytic AMPs with membranes in coarse-grained molecular dynamics simulations. The binding is characterized by the distribution and time evolution of backbone–phosphate (BB–PO_4) minimum distances, where BB represents the backbone beads of the AMPs, and PO_4 represents the phosphates of lipid molecules. **a**. Average BB–PO_4 minimum distances over the final 1 μs for 514 out of the 516 membrane-lytic AMP dataset. Each blue dot represents the time-averaged value for a single system. **b**. Time evolution of BB–PO_4 minimum distances for ten representative AMP systems during the final 1 μs, demonstrating that these peptides are stably bound to the membrane surface. **c**. Average BB–PO_4 minimum distances over the final 1 μs for the two outlier systems (AMP422 and AMP493), exhibiting significantly larger average BB–PO_4 distances. **d**. Time evolution of BB–PO_4 minimum distances for AMP422 and AMP493 over 5 μs trajectories, demonstrating that these two peptides could not stably bind to the membrane surface.

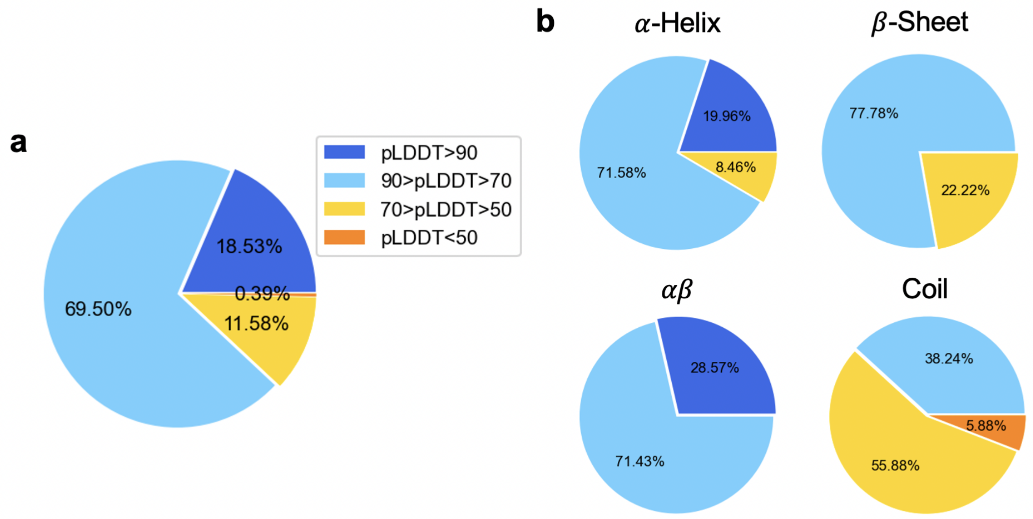

Figure S3: The confidence of 516 AlphaFold-predicted peptide structures. **a**. pLDDT distribution of 516 peptides. **b**. pLDDT distribution of peptides in different secondary structures.

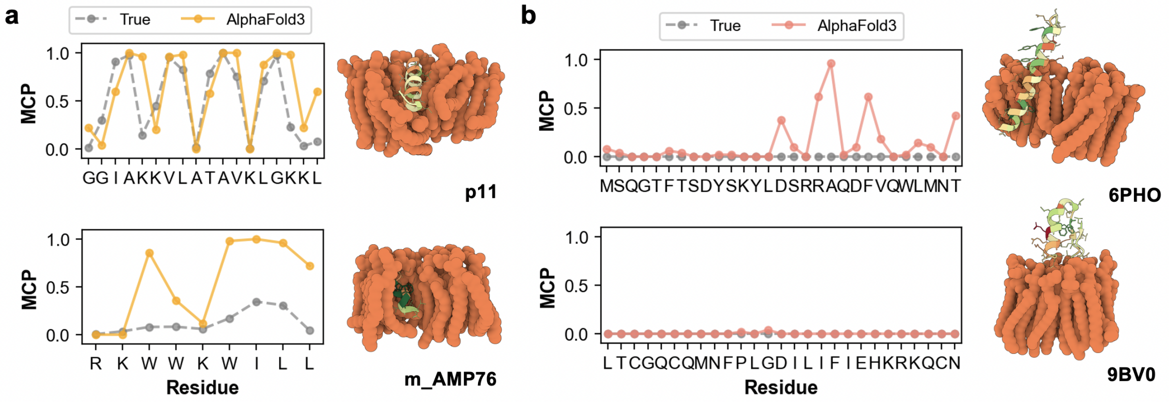

Figure S4: Case studies of AlphaFold3 predicted MCPs. **a**. AlphaFold3 predicted values, true MCP values from MD of two membrane-lytic AMPs with lipids, alongside their representatives of AlphaFold3 predicted structures. **b**. AlphaFold3 predicted values, true MCP values with all zeros of two soluble non-AMPs with lipids, alongside their representatives of AlphaFold3 predicted structures. Hydrophilic residues of peptides were colored in shades of orange and hydrophobic residues in shades of green.

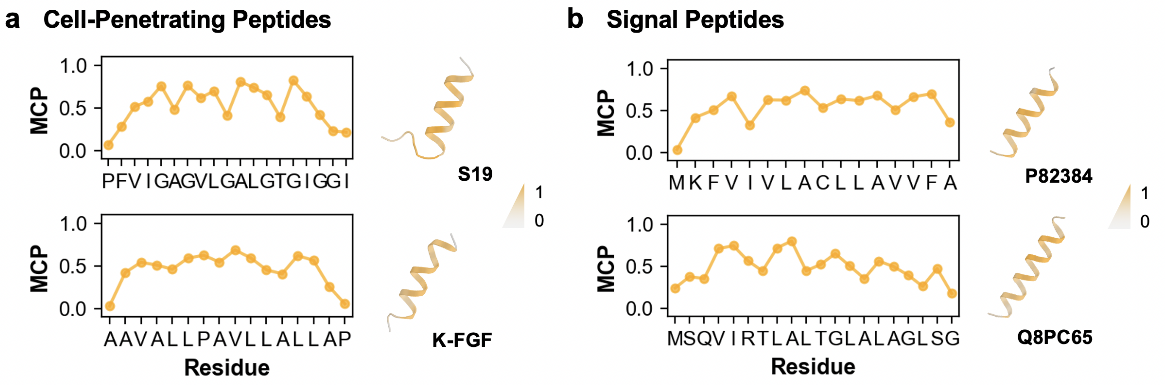

Figure S5: Case studies of PepMCP on other membrane-active peptides. **a**. PepMCP predicted values and structures of two cell-penetrating peptides. **b**. PepMCP predicted values and structures of two signal peptides. Structures were predicted using AlphaFold2 and colored with predicted MCP values.

Supplementary Tables

Table S1: Comparison of PepMCP models trained on residue-level and sequence-level split data on the external test set. Bold values indicate the best performance for that metric.

Task	Metric	Residue-level split	Sequence-level split
Regression	Spearman	0.7182	**0.7300**
	Pearson	**0.8226**	0.8191
	R^2	**0.6750**	0.6704
	RMSE	**0.1531**	0.1542
Classification	AUC	**0.9277**	0.9258
	AUPR	0.8606	**0.8855**
	Accuracy	**0.9000**	0.8625
	Precision	**0.9333**	0.9259
	Recall	**0.8235**	0.7352
	F1	**0.8750**	0.8196

Table S2: Comparison of the MCP threshold value for the classification task of PepMCP. Bold values indicate the best performance for that metric.

Threshold	Accuracy	Precision	Recall	F1
0.1	0.8875	0.8571	**0.8824**	0.8695
0.2	**0.9000**	**0.9333**	0.8235	**0.8750**
0.3	0.8125	0.9130	0.6176	0.7368

Table S3: Comparison of PepMCP model with DREAMM and PMIpred on the external test set. Bold values indicate the best performance for that metric.

Task	Model	Accuracy	Precision	Recall	F1
Residue-level classification	PepMCP	**0.9039**	**0.7054**	**0.8437**	**0.7684**
	DREAMM	0.4800	0.2449	0.7523	0.3695
Sequence-level classification	PepMCP	**0.9000**	**0.9333**	**0.8235**	**0.8750**
	PMIpred	0.7250	0.7727	0.5000	0.6071

Table S4: Comparison of the CD-HIT threshold of the membrane-lytic AMP dataset to train PepMCP. Results are reported using 5-fold cross validation (average $\pm$ standard deviations) or on the external test set with residue-split data. Bold values indicate the best performance for that metric.

Threshold	#MemAMPs	Spearman ↑	Pearson ↑	R^2 ↑	RMSE ↓
On 5-fold cross validation					
90%	516	0.8016 ± 0.0029	**0.8762 ± 0.0060**	**0.7664 ± 0.0107**	**0.1258 ± 0.0029**
80%	455	0.7956 ± 0.0021	0.8674 ± 0.0038	0.7441 ± 0.0113	0.1330 ± 0.0029
70%	406	0.7995 ± 0.0030	0.8734 ± 0.0023	0.7534 ± 0.0099	0.1352 ± 0.0027
60%	360	**0.8022 ± 0.0029**	0.8586 ± 0.0068	0.7297 ± 0.0218	0.1385 ± 0.0054
50%	261	0.7944 ± 0.0043	0.8270 ± 0.0104	0.6601 ± 0.0182	0.1618 ± 0.0043
On external test set					
90%	516	0.7182	**0.8226**	**0.6750**	**0.1531**
80%	455	0.7177	0.8094	0.6386	0.1615
70%	406	**0.7193**	0.8014	0.6319	0.1630
60%	360	0.6935	0.8036	0.6400	0.1612
50%	261	0.6874	0.7698	0.5877	0.1725

Table S5: Comparison of the length threshold of PepMCP's training dataset. Results are reported using 5-fold cross validation (average $\pm$ standard deviations) or on the external test set with residue-split dataset. Bold values indicate the best performance for that metric.

Length	#MemAMPs	Spearman ↑	Pearson ↑	R^2 ↑	RMSE ↓
On 5-fold cross validation					
51	516	0.8016 ± 0.0029	**0.8762 ± 0.0060**	**0.7664 ± 0.0107**	**0.1258 ± 0.0029**
40	481	0.8510 ± 0.0067	0.8664 ± 0.0083	0.7313 ± 0.0466	0.1575 ± 0.0130
30	406	**0.8677 ± 0.0029**	0.8635 ± 0.0105	0.7276 ± 0.0394	0.1710 ± 0.0122
On external test set					
51	516	**0.7182**	**0.8226**	**0.6750**	**0.1531**
40	481	0.6964	0.8102	0.6537	0.1581
30	406	0.6623	0.7714	0.5699	0.1762
